# Biomechanical stability of short versus long proximal femoral nails in osteoporotic subtrochanteric A3 reverse-oblique femoral fractures: a cadaveric study

**DOI:** 10.1007/s00402-022-04345-0

**Published:** 2022-01-21

**Authors:** Christoph Linhart, Manuel Kistler, Adrian C. Kussmaul, Matthias Woiczinski, Wolfgang Böcker, Christian Ehrnthaller

**Affiliations:** Department of Orthopaedics and Trauma Surgery, Musculoskeletal University Center Munich (MUM), University Hospital, LMU Munich, Marchioninistr 15, 81377 Munich, Germany

**Keywords:** Subtrochanteric fracture, PFNA, Reverse-oblique, Proximal femur, Geriatric trauma, Biomechanics femur

## Abstract

**Purpose:**

Due to the demographic change towards an older society, osteoporosis-related proximal femur fractures are steadily increasing. Intramedullary nail osteosyntheses are available in different lengths, where the field of application overlaps. The aim of this study was to investigate whether subtrochanteric fractures can also be treated stably using a short femoral intramedullary nail in cadaveric bones.

**Methods:**

A short PFNA and a long PFNA were implanted in both seven artificial bones and osteoporotic human specimens. A standardized AO 31-A3 (reverse-oblique) fracture was placed in the specimens with a lateral fracture spur 2 cm proximal to the distal locking screw (short PFNA) and embedded. The simulated iliotibial tract was preloaded to 50 N. The force was applied at 10 mm/min up to a force of 200–800 N (artificial bones) and 200–400 N (human specimens). The dislocation of the fracture gap, the axial bone stiffness of bone construct and the force curve of the tractus iliotibialis were measured.

**Results:**

There is no difference in the use of a short versus long PFNA in terms of stiffness of the overall construct and only a slight increase in dislocation in the fracture gap results with short PFNA compared to a long intramedullary nail.

**Conclusion:**

In summary of the available literature, the present study supports the thesis that there is no clinical difference between long versus short nails in A3 femur fractures. Furthermore, the present study defines a safe biomechanical range of fracture extension above the locking screw of the short intramedullary nail.

**Level of Evidence:**

III

## Introduction

The incidence of proximal femur fractures caused by osteoporosis increases steadily—not least because of the demographic changes of the elderly society [1]. In Germany, the incidence rate is 90/100,000 inhabitants [2, 3]. Thus, suffering a proximal femur fracture is a life-changing event for older patients. Subsequent impairment, sometimes permanent, of activities of daily living and independence is common and mortality rates of up to 20% can be observed in the first year after the fracture [4, 5]. The annual number of patients with proximal femur fractures, with an increasing incidence of pertrochanteric femur fractures, is expected to rise to as many as 6.3 million per year by 2050 due to the aging population [6, 7].

Today, minimally invasive intramedullary nailing is considered as the treatment of choice for pertrochanteric femoral fractures based on biomechanical advantages of an intramedullary force carrier compared to an extramedullary fracture treatment [8–10]. Different implants are available for intramedullary fixation, e.g., proximal femoral nail antirotation (PFNA, DePuy Synthes^®^ Inc., West Chester, USA) or gamma nail (Stryker^®^, Kalamazoo, USA). The implants work on the same basic principle: an intramedullary nail extends from the greater trochanter down into the femoral shaft and one or two components (screws or blades) inserted from the nail through the femoral neck into the femoral head. The PFNA has a specially designed spiral blade preventing rotation and results in compression of the cancellous structures in the osteoporotic bone.

In recent years, research has focused mainly on avoiding implant failure [11, 12]. Maintaining the correct tip-apex distance (TAD) has been established as a safe way to avoid a cut-out at less than 25 mm in both radiographic planes [13]. Another practical aspect for the correct positioning of the implant in the femoral neck is the so-called “center–center” position in both X-ray planes, which is currently considered as the most favorable position by most authors [13, 14].

The classification into stable and unstable fracture patterns was also adopted from trochanteric fractures in the revised AO/OTA classification. Here, the fracture entities AO/OTA 31-A1.1-3 are considered stable fracture morphologies with wide acceptance of intramedullary fracture treatment e.g. by a short intramedullary proximal femur nail. The fracture entities AO/OTA 31-A2.2-3.3, on the other hand, are described as unstable and do not underly a general recommendation for surgical fracture treatment.

The optimal treatment of subtrochanteric femoral fractures (AO/OTA 31-A3) is discussed highly controversial in current literature. According to the manufacturer, high subtrochanteric fractures represent an indication for the short nail, whereas “more distal subtrochanteric fractures” are contraindicated. This vague statement routinely leads to the use of a long nail in a majority of subtrochanteric fractures not knowing where the exact border should be drawn. Intramedullary treatment with a long PFNA is often preferred compared to a short PFNA because of a purported higher stability. In addition, an open reduction with implantation of a wire cerclage is commonly described in the literature—however clinical studies show no advantage of a long compared to a short nail [15–18]. Until now, a systematic biomechanical comparison of stability after long or short intramedullary nail osteosynthesis of subtrochanteric fractures is lacking [19, 20]. The high clinical relevance of the implant choice results not least from the prolonged surgery time, the extensive blood loss and the pronounced soft tissue trauma when choosing a long intramedullary implant in stable as well as unstable pertrochanteric femur fractures [15, 16, 21, 22].

The aim of this study is to provide the biomechanical basis for decision-making on the optimal choice of implant for subtrochanteric femoral fractures with the hypothesis that surgical treatment with a short intramedullary nail is feasible for these fractures, depending on the fracture extension at the lateral cortex.

## Materials and methods

Our study includes three biomechanical sub-tests with initial testing on the artificial bone (part I) and final testing on human cadaver (part II). After obtaining approval from our institutional review board (no. 8–030), a total of 18 biomechanical artificial bones were used for the first two sections of this biomechanical study (sawbones Europe AB, malmoe, sweden, femur, PCF 17, medium, 4th generation Composite). For the third part cadaver femora were used and for this purpose 14 fresh-frozen human femora were purchased commercially from Science Care (Phoenix, AZ, USA). The femora were frozen at − 24 °C and thawed at room temperature 24 h before the planned testing. To ensure osteoporotic status of the bones, only bones from female donors older than 75 years were acquired. To increase comparability and reduce bias, only matched pairs of femora were used. The uniformity of the experimental groups was further optimized by measuring bone mineral density (qCT) and then assigning the sample to the two experimental groups.

**Fig. 1:**
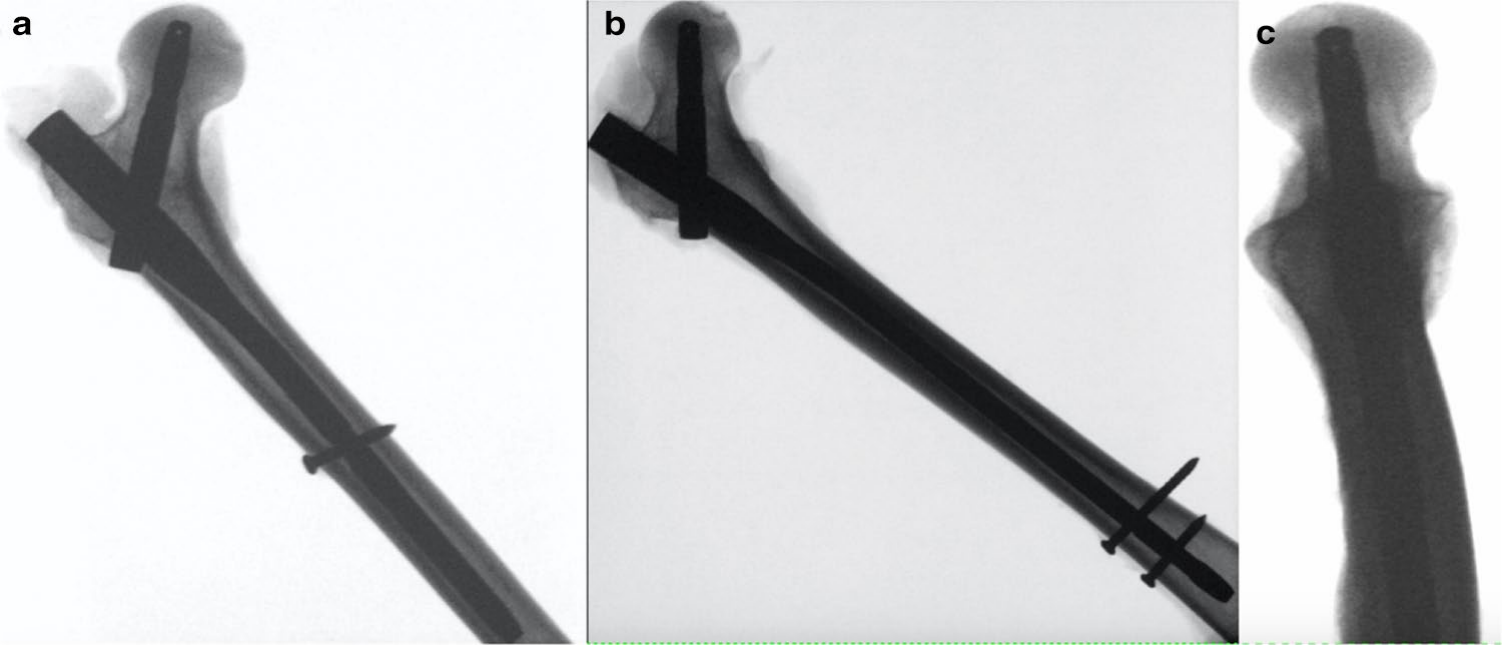
X-ray image of the correct implantation of the short (**a**) and long (**b**) PFNA in the cadaver bone in a.p. **a** + **b** and axial (**c**) view, taking into account the correct position of the intramedullary nail and the femoral neck blade

**Fig. 2:**
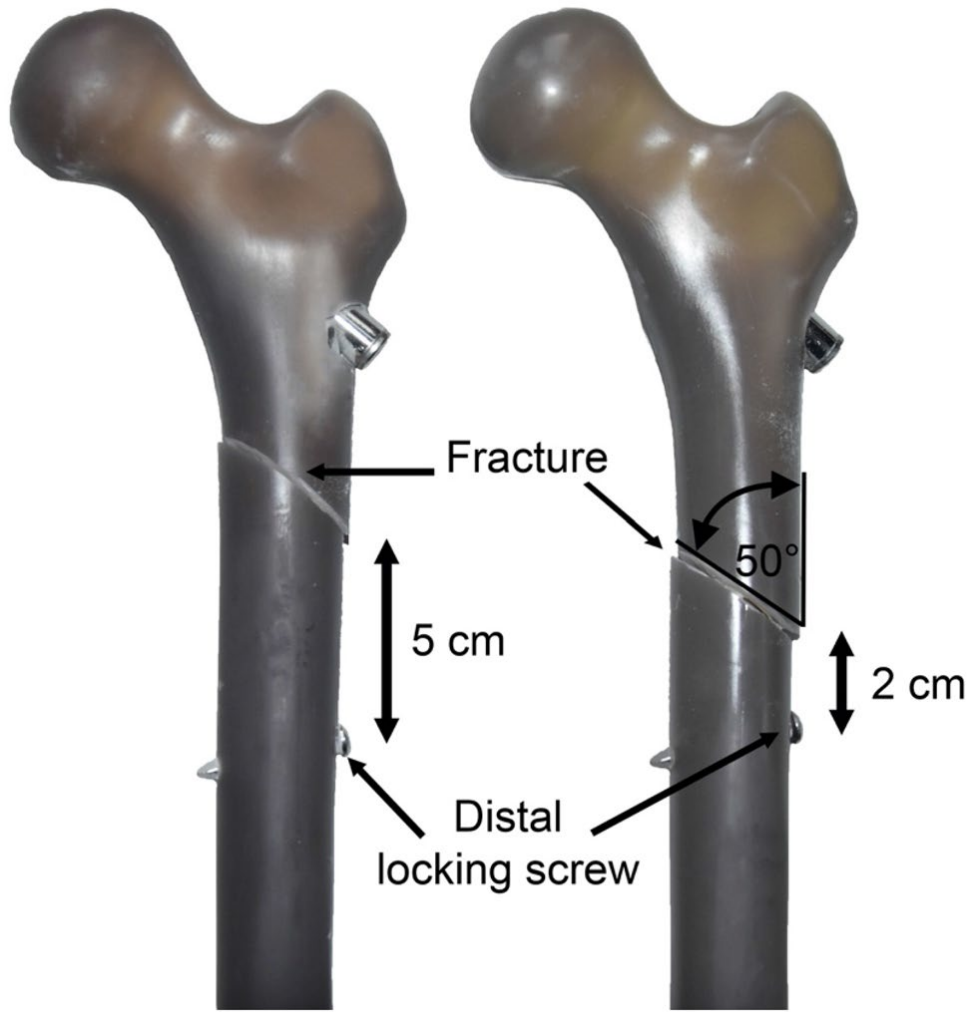
Photograph of biomechanical specimens after implantation of a short PFNA and "reverse-oblique" osteotomy on different locations in the artificial bone

The surgical technique of PFNA intramedullary nail implantation was performed according to the manufacturer's instructions (Fig. 1). For the purpose of minimally invasive implantation and standardization, the original manufacturer's instrumentation was used. A short (10 × 240 mm) and a long (10 × 320 mm) PFNA was used. The fracture was set standardized according to an AO 31-A3 fracture 50° oblique in terms of a "reverse-oblique" fracture. After successful implantation of the PFNA, the fracture was marked on the intact bone. The angle was determined from the shaft axis. The osteotomy was carefully performed with a hand saw without damaging the nail. For pretesting, four artificial bones were instrumented using a short PFNA and two fracture variants were examined with increasing extension of the lateral fracture extension in direction of distal. After inducing a lateral fracture 5 cm proximal to the distal locking screw, the fracture was displaced to a distance of 2 cm to the distal locking screw (Fig. 2). These pretests indicated no difference in fracture gap dislocation, axial bone stiffness and force distribution of the iliotibial tract between the different locations of the fracture (2 cm and 5 cm) Table 1. The largest movement at 200 N at a distance of 5 cm was 1.00 ± 0.41 mm while the fracture at 2 cm moved 0.98 ± 0.25 mm. Even at 800 N, there were no significant differences between the locations of the fracture 5 cm (2.32 mm ± 0.96 mm) and 2 cm (1.64 mm ± 0.14 mm). Thus results, the most distal fracture configuration with still acceptable stability compared to the most proximal fracture line (> 60%) was then used for part I and II of the study, this was 2 cm proximal to the distal locking screw using the short nail. For the second part, either a long or a short intramedullary nail was implanted in each of the seven artificial bones and the biomechanical stability was examined. The preceding tests on the artificial bone thus formed the foundation for the third part, the investigation on the cadaver femur. In this context, the biomechanical stability of the selected fracture entity after short or long intramedullary nail osteosynthesis was investigated on seven cadaver femora each.

**Table 1:** Overview of mean values and standard deviation for dislocation, axial bone stiffness and force of the iliotibial tract as a function of test loads in cadaveric and artificial bones

Bone	Dislocation fracture gap in mm			
	Short PFNA		Long PFNA	
	Mean (*n* = 7)	SD	Mean (*n *= 7)	SD
Artificial 200 N	0.83	0.16	0.99	0.16
Artificial 400 N	0.98	0.27	1.16	0.18
Artificial 600 N	1.31	0.29	1.41	0.35
Artificial 800 N	1.58	0.42	1.48	0.47
Cadaveric 200 N	1.01	0.17	0.76	0.12
Cadaveric 400 N	1.49	0.30	1.04	0.26

	Axial bone stiffness in N/mm			
	Short PFNA		Long PFNA	
	Mean (*n* = 7)	SD	Mean (*n* = 7)	SD
Artificial 200 N	50.22	12.21	43.22	14.59
Artificial 400 N	50.65	7.60	43.21	9.43
Artificial 600 N	50.97	6.10	40.19	5.06
Artificial 800 N	51.10	4.98	42.36	5.20
Cadaveric 200 N	28.43	4.67	36.08	8.45
Cadaveric 400 N	34.18	6.27	32.26	3.41

	Force in the iliotibial tract in N			
	Short PFNA		Long PFNA	
	Mean (*n* = 7)	SD	Mean (*n* = 7)	SD
Artificial 200 N	307.52	26.14	302.07	14.40
Artificial 400 N	579.43	25.41	598.04	28.42
Artificial 600 N	892.63	49.83	964.05	44.18
Artificial 800 N	1211.64	59.73	1287.86	174.22
Cadaveric 200 N	342.40	24.36	337.50	16.52
Cadaveric 400 N	710.80	34.67	699.80	34.12

To ensure the quality of the osteosynthesis, the following criteria were assessed by means of an X-ray in two planes during implantation of the nails (Fig. 1): correct entry of the PFNA at the tip of the greater trochanter; correct length of the PFNA (10 mm subcortical to the joint line); "center-center" position of the blade (centrally placed in two planes); “tip-apex" distance of less than 25 mm from both planes summed (blade tip-joint line distance); bicortical position of the distal locking screw; no penetration of the articular surface. If a restoration did not met the above-mentioned criteria, the specimen was excluded from the study.

### Biomechanical testing

**Fig. 3:**
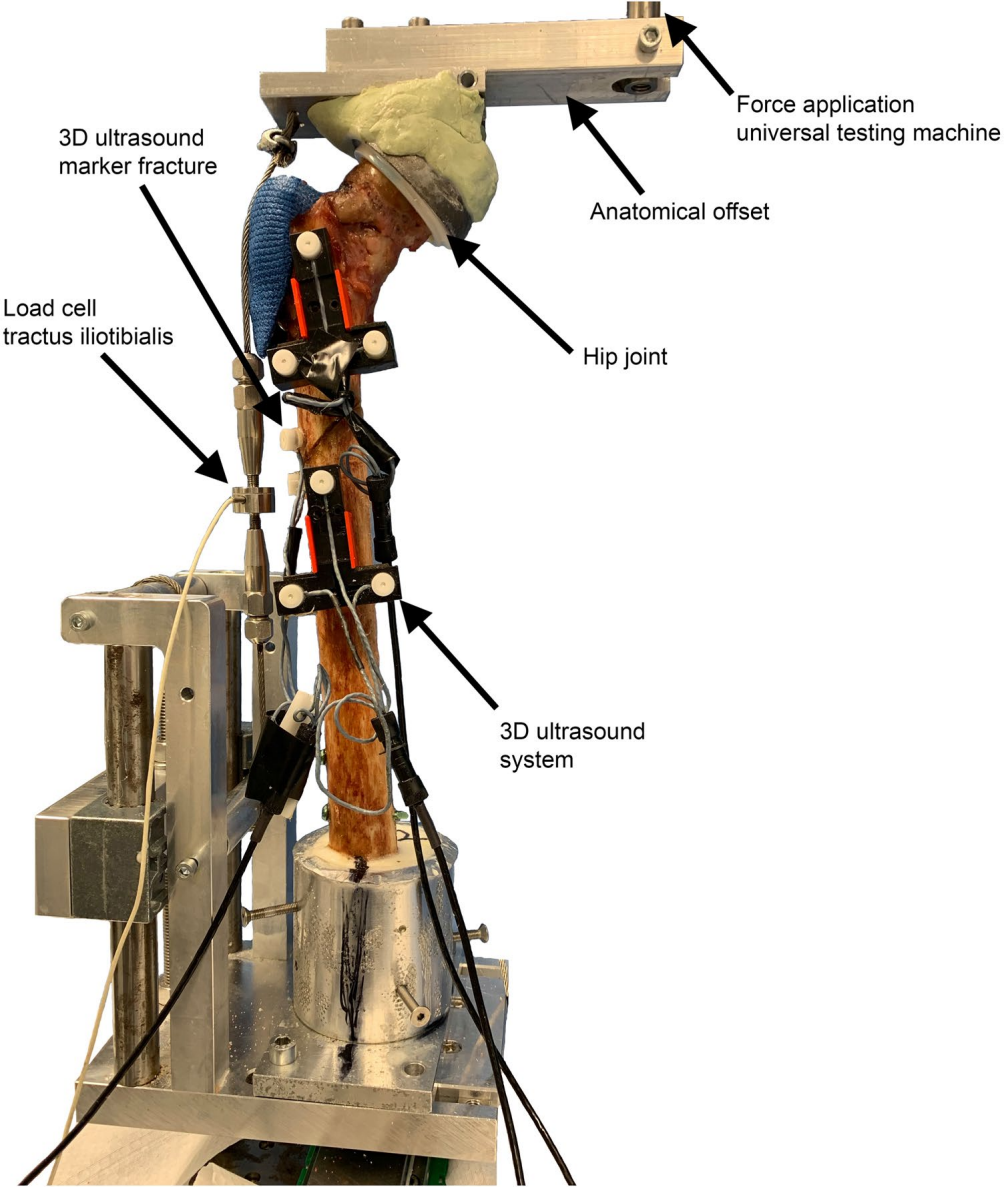
Photograph of the biomechanical test setup for force application in the universal testing machine with simulated iliotibial tract embedded in a metal pot

All bones were tapered at the distal end and embedded under a 6° valgus position in metal pots using a polyurethane resin (RenCast^®^ FC 52/53 Isocyanat; FC 53 Polyol, Huntsman Corporation, The Woodlands, Texas, USA) as in former studies [23]. The function of the iliotibial tract was simulated with a 3 mm steel cable that becomes continuously stretched with increasing load (Fig. 3). To measure the force characteristic of the simulated tractus iliotibialis during force application of the bone, a 5 kN load cell (8417–6005, Burster Praezisionsmesstechnik GmbH & Co. KG, Gernsbach, Germany) was implemented in the wire rope. A program was created in LabVIEW 2014 (Version 14.0.1, National Instruments, Austin, USA) to calibrate the sensor and collect the force data. Sensor calibration was done using the universal testing machine. Specified tensile and compressive forces in the range from − 200 N (compressive force) to + 1500 N (tensile force) were applied to the force sensor in 100 N increments. A 3D ultrasound system (Zebris CMS 20, Zebris Medical GmbH, Isny, Germany) was used to track the scope of 3D movement of the bone segments to measure the displacement of the fracture gap in three dimensions in millimeter. Depending on the application, the system has a resolution of 1/10 mm–1/100 mm with a measurement error of 0.25%. These ultrasound markers were screwed in place by additively manufactured holder or in some special cases directly to the bone with hot glue. All tests were performed in an electrodynamic universal testing machine (ElectroPuls™ E10000, Instron, Norwood, USA). The simulated tractus iliotibialis was slightly preloaded before loading until an effective joint force of 50 N was applied on the testing machine. Afterwards biomechanical testing of the instrumented specimens was Afterwards biomechanical testing of the instrumented specimens was performed under displacement-controlled force application at 10 mm/min up to effective joint loads of 200 N, 400 N, 600 N and 800 N for the artificial bones and 200 N and 400 N for the human specimens without creating a failure. All load procedures were repeated three times to avoid possible settling of the implant. Biomechanical stability was assessed by measurement the amount of fracture displacement and axial stiffness of bone construct (N/mm) of the instrumented specimen. Stiffness was defined as the resistance of the bone construct against elastically deformation in response to an applied load without considering the geometry of the cross section. Additionally, force distribution of the iliotibial tract (N) during force application of the bones were measured as a secondary parameter for dislocation due to the expected tension release following fragment dislocation. The entire experimental setup is shown in Fig. 3 with a human cadaver.

### Statistical analysis

For data collection and analysis, the software MATLAB (R2020a, MathWorks Inc., Natick, USA) and Excel were used.

The Shapiro–Wilk test was used to evaluate the data for normal distribution. The *t* test for unpaired samples was used between long and short PFNA to compare the fracture gap dislocation, axial bone stiffness, and force distribution of the iliotibial tract. Differences with a *p* value < 0.05 were defined as significantly different. All statistical analyses were performed with GraphPad Prism 8 (GraphPad Software, Inc., La Jolla, USA). The mean and standard deviation were presented for all graphs.

## Results

### Comparison short vs long pfna—artificial bone—part I

The dislocation of the fracture gap showed no significant difference between short and long PFNA in the artificial bone. The movement resulted values between 0.83 ± 0.16 mm (200 N) and 1.58 ± 0.42 mm (800 N) for the short PFNA and 0.99 ± 0.16 mm (200 N) and 1.48 ± 0.47 mm for a long PFNA. Therefore, this show that the dislocation at loads of 800 N is 7% lower with a long PFNA than with a short nail. The axial bone stiffness of the artificial bone with a short PFNA was significantly higher at testing forces of 600 N (27%; *p* = 0.004) and 800 N (21%; *p* = 0.008) than a long PFNA.

**Fig. 4:**
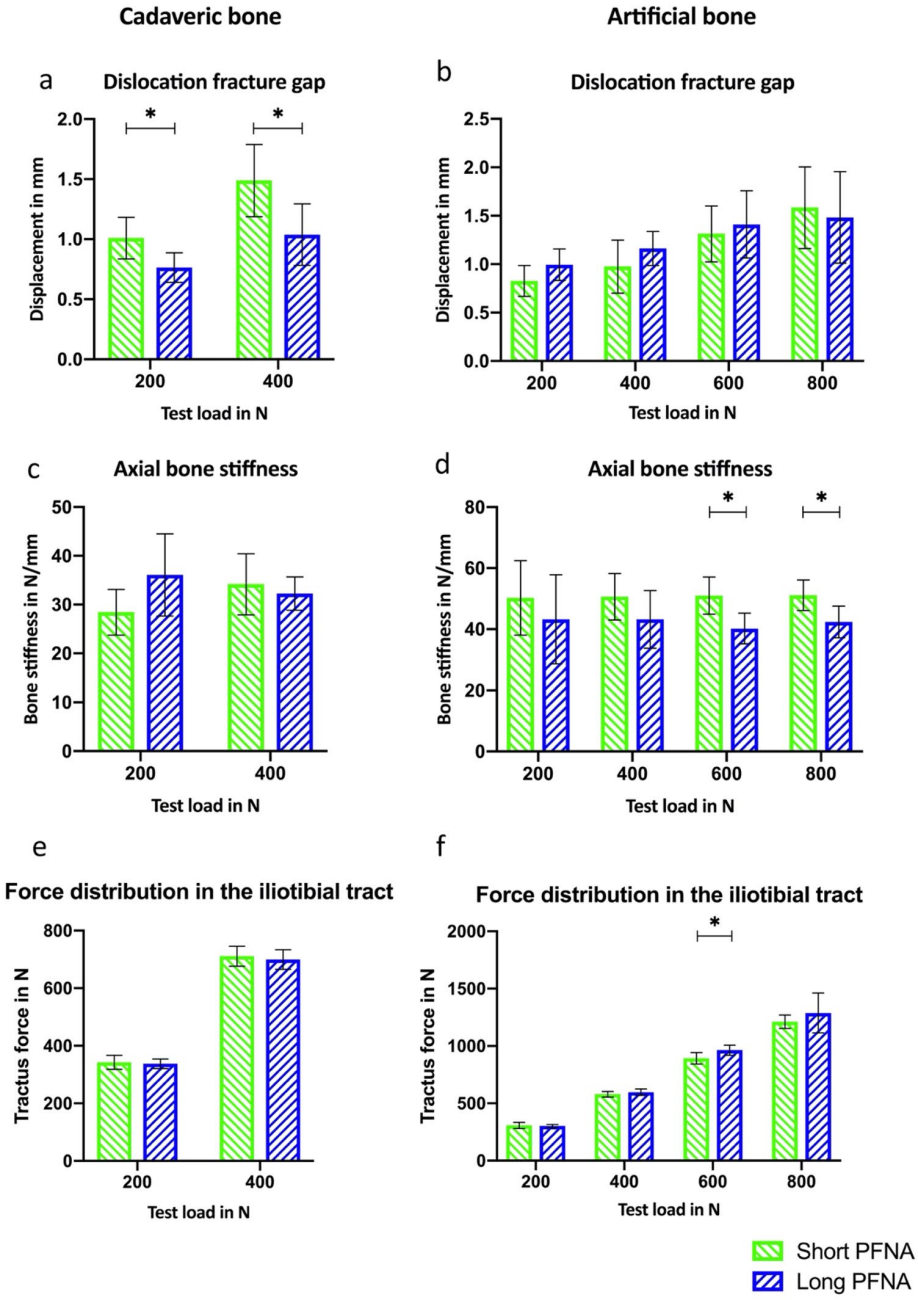
Dislocation (**a** and **b**), axial stiffness (**c** and **d**) and force of the iliotibial tract (**e** and **f**) in cadaveric and artificial bones. *Describes significance

The force of the iliotibial tract in the artificial bone showed also no significant change between long and short PFNA. Only a test load of 600 N displayed a significantly higher tractus iliotibialis force of 7% with a long PFNA (*p* = 0.02). For all other test forces the differences in iliotibial tract were maximum 2–6%. These results are shown in Fig. 4b, d and f.

### Comparison short vs. long pfna—cadaveric bone—part II

The results with the cadaveric bones indicated significantly lower dislocation with a long PFNA at a force of 200 N (*p* = 0.01) and 400 N (*p* = 0.01). The short PFNA moved 1.01 ± 0.17 mm to 1.49 ± 0.30 mm while the long PFNA showed smaller movements from 0.76 ± 0.12 mm to 1.04 ± 0.26 mm. This means that the largest absolute difference in dislocation values after treatment with a long PFNA were lower by 0.25 mm (200 N) and 0.45 mm (400 N). No significant difference in bone stiffness were observed. The maximum difference was 21% between the two intramedullary nails. The force of the iliotibial tract in the cadaver preparation also showed no significant changes (1–2%) between long and short PFNA. The results from part II are also shown in Fig. 4a, c and e.

## Discussion

Main finding of this study was to demonstrate for the first time a biomechanical decision-making basis for stabilization of proximal subtrochanteric reverse-oblique A3 femur fractures using an intramedullary fixation device.

We were able to demonstrate, that there is no difference in the use of a short versus long PFNA in terms of stiffness of the overall construct and only a slight millimeter increase in mobility in the fracture gap results with short PFNA compared to treatment using a long intramedullary force carrier. Although statistically significant, clinical relevance of an increase of fracture gap displacement in short PFNA compared to long PFNA below one millimeter remains questionable.

Up to now, there has been little or no evidence regarding subtrochanteric, reverse-oblique femoral fractures and, as a result, no instructions as to which osteosynthesis form should be given preference exist [15, 16, 19, 21].

Consequently, to obtain enough stability, it is common practice to give preference to the use of a long intramedullary force carrier even in cases of subtrochanteric fractures that are associated with only minor dislocation and fracture extension.

While open reduction and use of a long femoral intramedullary nail is inevitable in cases of severe dislocation, long spiral and segmental fractures extending into the diaphysis, the question arises as to how to proceed with less severely dislocated fractures without a large zone of comminution and an extension above the locking screw of a short intramedullary nail.

The question of the surgical procedure is by far not only a technical question, because the use of a short proximal femoral intramedullary nail differs significantly from that of a long intramedullary nail in many aspects. First, the use of a long intramedullary nail usually necessitates reaming of the femoral medullary canal so that the long intramedullary nail has adequate stability and can safely pass through the isthmus of the femur with risk minimization for femur shaft fractures. It is known that reaming of the medullary canal alone is capable of triggering a systemic inflammatory reaction with the risk for the development of a systemic inflammatory response syndrome as a result of a second hit in regard to the damage control principle [24, 25]. Furthermore, the use of a long intramedullary nail is often combined with open reduction and cerclage wiring of the main fragments. As a consequence of the greater incisions, blood volume loss increases together with surgical duration and fluoroscopy time [15, 16, 19, 21]. Additionally, open reduction inherits the risk of injury to vascular and nerve structures on the medial side of the femur, ultimately increasing the second hit in this highly vulnerable ortho-geriatric patient population. Only recently it has been shown in a geriatric patient collective, that with increasing instability of proximal femur fractures, clinical outcome worsens in respect to functional outcome, rehabilitation potential, loss of self-care potential and ultimately mortality [26]. Although it is not clear which part of the poor outcome is fracture-specific and which is care-specific, it is obvious that elderly, multimorbid patients in particular benefit from the gentlest possible surgical procedure.

It has been highlighted in several studies using mobility monitoring, that early mobilization is among the most important factors with influence to outcome in ortho-geriatric patients [27]. Optimized surgical treatment, omitting unnecessary long proximal femoral implants, might therefore lead to faster early mobilization due to reduced postoperative pain and might ultimately improve long-term outcome. From this it becomes apparent, that the decision to use a long intramedullary implant should be made carefully based on scientific data.

Unfortunately, there are not many studies on this subject that can help the treating surgeon in the decision-making process. While no clinical advantages exist in terms of stability, healing time and complications of long versus short nails in pertrochanteric fractures, increased blood loss and duration of the surgical procedure for long nails have been demonstrated here [15, 16].

For A3 fractures, the scientific evidence is even lower. The authors are aware of only one clinical study comparing short and long nails in reverse-oblique A3 fractures. Here, no significant differences between long and short nails could be found in any parameter investigated [22]. Unfortunately, the number of cases was very small, limiting the validity of this study. Another limitation is that no examination of the extension of the fracture towards the isthmus was performed.

Matching the results of the above-mentioned clinical study, a meta-analysis on the question whether to use short or long intramedullary nails best for proximal femur fractures was also unable to show any significant advantage of long nails [20].

Our study provides both strengths and limitations. A limitation is that the study was carried out in a model without soft tissues and fractures were induced by sawblades, which is not the case in the clinical setting. However, this method ensures the reproducibility of the fracture patterns across specimens, which is paramount for precisely addressing the hypothesis of this study. The relatively small sample size is a drawback that is based on the limited availability of fresh-frozen human femoral bones and is comparable to that of previous studies. The strengths of the study are the reproducible fracture patterns and the standardized measurement of the dislocation. In addition, after the preliminary tests on artificial bones, this study was carried out exclusively on human bones, which represents a much more realistic test setup than numerous published studies that were only carried out on artificial bones.

## Conclusion

In summary of the available literature, the present study supports the thesis that there is no clinical difference between long versus short nails in A3 femur fractures. Furthermore, the present study defines a safe range of fracture extension above the locking screw of the short intramedullary nail, which allows for equal biomechanical results in reference to the long intramedullary nail. Further clinical studies should be obtained to confirm our results defining precise indications for the use of a short proximal femur nail.
